# Mindfulness-Based Interventions for Mental Health Outcomes in Frontline Healthcare Workers During the COVID-19 Pandemic: A Randomized Controlled Trial

**DOI:** 10.1007/s11606-025-09529-z

**Published:** 2025-05-19

**Authors:** Marieke Arts-de Jong, Dirk E. M. Geurts, Philip Spinhoven, Henricus G. Ruhé, Anne E. M. Speckens

**Affiliations:** 1 Department of Psychiatry, Radboud University Medical Centre, Nijmegen, The Netherlands; 2 Donders Centre for Medical Neuroimaging, Donders Institute for Brain, Cognition and Behaviour, Radboud University Nijmegen, Nijmegen, The Netherlands; 3 Institute of Psychology, Leiden University, Leiden, The Netherlands

**Keywords:** mindfulness-based intervention, MBSR, COVID-19 pandemic, frontline healthcare workers, mental health

## Abstract

**Background:**

The COVID-19 pandemic significantly impacted the mental health of frontline healthcare workers (HCWs), but solid evidence on psychological interventions for HCWs remains limited.

**Objective:**

Whether an adjusted therapist-assisted Mindfulness-based Stress Reduction group intervention (adjusted MBSR) is superior to a minimal self-guided mindfulness-based intervention (self-guided MBI) in improving mental health of HCWs during the COVID-19 pandemic.

**Design:**

Randomized controlled trial.

**Participants:**

201 frontline HCWs (47 physicians, 120 nurses, 34 supporting staff); enrollment between June 2020 and September 2021.

**Interventions:**

A 4-week adjusted MBSR with eight biweekly 1.5-h sessions; or a 4-week self-guided MBI with 24 mindfulness/compassion exercises.

**Measures:**

Primary outcome was the Patient Health Questionnaire – Somatic, Anxiety and Depressive Symptom Scales (PHQ-SADS) at 6-month follow-up. Secondary outcomes included posttraumatic symptoms, insomnia, alcohol use, repetitive negative thinking, mental well-being, posttraumatic growth, mindfulness, and self-compassion at post-intervention and 3- and 6-month follow-up.

**Key Results:**

At 6-month follow-up, the adjusted MBSR was not superior to the self-guided MBI (mean difference (SE) PHQ-SADS, 0.23 (1.03), *P*=0.82). Both interventions showed similar within-group improvement in PHQ-SADS (Cohen’s *d* between baseline and 6-month follow-up: adjusted MBSR −0.78 (95% CI −1.07; −0.48), self-guided MBI −0.72 (95% CI −1.01; −0.43)). Secondary outcomes showed that symptom trajectories differed between groups for PHQ-SADS (intervention*time *F*(3, 420)=3.99, *P*=0.008), with greater reduction at post-intervention for adjusted MBSR, and posttraumatic growth (intervention*time *F*(3, 350)=5.32, *P*=0.001), with exclusive increase post-intervention in adjusted MBSR. Both interventions showed comparable significant within-group improvements on posttraumatic symptoms, insomnia, repetitive negative thinking, mental well-being, mindfulness, and self-compassion.

**Conclusions:**

The adjusted MSBR was not superior to the self-guided MBI; both were accompanied by significant reductions of depressive, anxiety, and somatic symptoms after 4 weeks of treatment which was sustained at 6-month follow-up. Further research is needed to investigate the possible role of MBIs to support HCWs involved in future healthcare crises.

**Trial Registration:**

ClinicalTrials.gov NCT04720404; onderzoekmetmensen.nl/en NL73793.091.20

**Supplementary Information:**

The online version contains supplementary material available at 10.1007/s11606-025-09529-z.

## INTRODUCTION

After the COVID-19 outbreak, evidence soon emerged about the significant impact of the pandemic on mental health of frontline healthcare workers (HCWs), resulting in a high prevalence of symptoms of depression, anxiety, insomnia, and posttraumatic stress.^1–3^ Longitudinal research revealed that working as a frontline HCW increased the risk of persistent depressive and posttraumatic stress related symptoms.^4,5^ This highlighted the urgency of mental support for frontline HCWs to reduce psychological symptoms, improve sustainability of healthcare provision, and prevent possible drop-out.^6^ Evidence-based mental health interventions to support COVID-19 frontline HCWs were needed,^7,8^ but relatively few RCTs on reducing psychological distress were conducted at the time or have been published since.^9–16^ Moreover, these interventions should be adapted to the crisis situation, such that these are acceptable, feasible, and scalable under difficult circumstances inherent to pandemics.

Mindfulness-based interventions (MBIs) are potent interventions with a broad evidence base in reducing psychological symptoms and increasing positive mental health.^17–21^ Participants are encouraged to improve self-awareness; adopt a non-judgmental attitude toward bodily sensations, thoughts, and emotions; and enhance self-care.^22^ The effectiveness of MBIs in reducing psychological symptoms has been demonstrated in both clinical^17^ and non-clinical^21^ populations. When applied in HCWs, MBIs have been demonstrated to decrease burnout, anxiety, depression, and psychological distress and increase well-being, mindfulness skills, and empathy.^19,20^ Moreover, a recent systematic review and meta-analysis suggests that both therapist-assisted and self-guided web-based MBIs may be effective in reducing symptoms of anxiety, depression, and stress among frontline HCWs during the COVID-19 pandemic.^23^ However, the main conclusion was that studies of higher quality with longer follow-up are necessary to substantiate the suggestion of efficacy.^23^

To support frontline HCWs during this pandemic, we set up an RCT with 6-month follow-up to investigate the effectiveness of a 4-week adjusted therapist-assisted Mindfulness-based Stress Reduction (MBSR) group intervention. The ethical review committee deemed it unethical to withhold a MBI mindfulness-based intervention from interested frontline HCWs, and insisted on some sort of active comparator as a waitlist control was not considered satisfactory. Furthermore, the committee argued that the proposed adjusted MBSR had sufficient indirect evidence to assume efficacy. Therefore, we could not include a treatment as usual control condition, and created a minimal intervention instead. We hypothesized that an adjusted therapist-assisted MBSR group intervention would be more effective to support frontline HCWs over a 6-month follow-up period than a 4-week minimal self-guided MBI.

## METHODS

### Design

**Figure 1:**
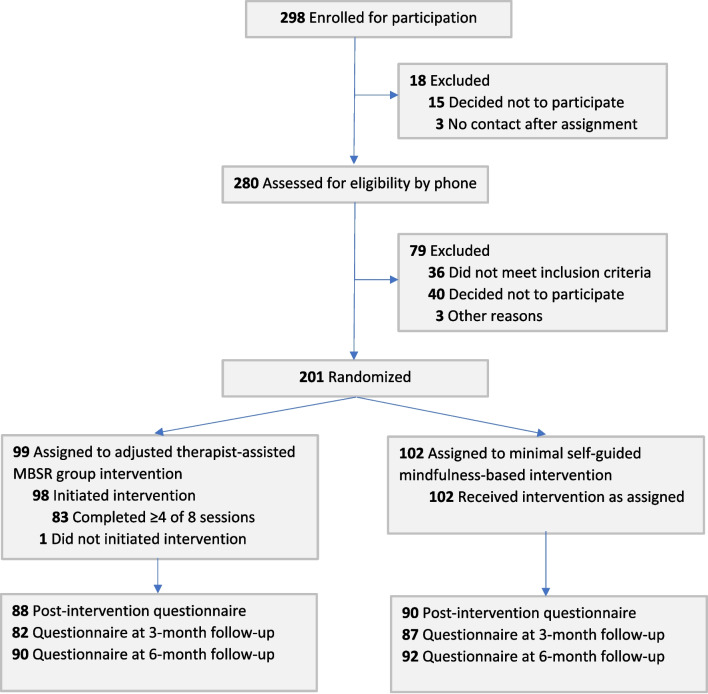
CONSORT flow diagram.

This nationwide two-armed, single-blinded, randomized controlled trial took place in The Netherlands from June 2020 to June 2022 during the COVID-19 pandemic. We used a superiority design to determine whether an adjusted therapist-assisted MBSR group intervention (adjusted MBSR) was more effective than a minimal self-guided MBI (self-guided MBI) in frontline HCWs. Participants were randomized with an allocation ratio of 1:1 using an Electronic Data Capture (EDC) program (Castor: https://www.castoredc.com). The study coordinator carried out the randomization when eligibility was confirmed and emailed the allocation to participants. Randomization was stratified by setting (hospital, nursing home, other), profession (physician, nurse, other), and prior 8-week mindfulness training (yes/no). A variable block randomization (block sizes 2 or 4) was used to ensure balanced groups. Cohorts were formed by participants randomized to both arms in which the interventions started simultaneously (almost monthly). Data were collected at baseline (T0), post-intervention at 4 weeks (T1), and at 3-month (T2) and 6-month (T3) follow-up using Castor EDC software. Participants in the self-guided MBI were offered the possibility to participate in an adjusted MBSR after completion of the study. For recruitment, randomization, and patient flow, see Fig. 1. We followed the Consolidated Standards of Reporting Trials (CONSORT) guideline.^24^

### Participants

Eligible participants were HCWs (18 years or older) *directly* involved in the acute care for patients with COVID-19 in the 4 months preceding enrollment. Exclusion criteria included insufficient comprehension of the Dutch language and inability to access the interactive video conferencing.

The executive boards of all general and university Dutch hospitals that provided acute care to COVID-19 patients based on “Zorgkaart Nederland”^25^ (*n*=66), nursing homes (*n*=22), and general practices and homecare services (*n*=10) were informed about this trial and asked to participate and distribute study information to COVID-19 HCWs in their organizations. Social media (LinkedIn) was also used to advertise this study. The study was coordinated by Radboud University Medical Center.

### Interventions

#### Adjusted Therapist-Assisted MBSR Group Intervention (adjusted MBSR)

The adjusted MBSR included the core components of the Mindfulness-Based Stress Reduction program^26^ with additional compassion exercises. This 4-week intervention consisted of eight 1.5-h sessions (i.e., twice a week) and was taught by two qualified mindfulness teachers with more than 15 years of mindfulness teaching practice. Participants received a personal workbook with information used during the training and had access to sound recordings of guided meditations. Daily mindfulness practice at home was strongly encouraged. The teacher recorded the number of sessions attended for each participant. Completers were defined as having attended four or more sessions.^27^ More detailed information per session is provided in eTable 1.

#### Minimal Self-guided Mindfulness-Based Intervention (self-guided MBI)

Participants in the self-guided MBI only received one brief document at the start of the intervention via email of a 4-week program with links to 24 instruction videos or audio files (six per week) of approximately 30 min with a short introduction of a theme, such as bodily awareness, and a guided mindfulness or compassion exercise. These files mainly consisted of previously recorded meditations specifically for COVID-19 HCWs. The program was constructed in such a way that it mirrored the structure of the adjusted MBSR program in terms of discussed themes and exercises offered. More detailed information and links to the videos are provided in eTable 1.

### Outcome Measures

The primary outcome was a composite measure of depressive, anxiety, and somatic symptoms as assessed with the Patient Health Questionnaire – Somatic, Anxiety and Depressive Symptom Scales (PHQ-SADS)^28^ at 6-month follow-up. This self-report measure combines three questionnaires: the 9-item PHQ (PHQ-9) (range 0–27),^29^ the 7-item Generalized Anxiety Disorder (GAD-7) (range 0–21),^30,31^ and the 15-item PHQ (PHQ-15) (range 0–30).^32^

Previous studies using confirmatory factor analysis revealed sufficient unidimensionality to support the use of the total score of the Patient Health Questionnaire – Anxiety and Depression Scale (PHQ-ADS) as a composite measure of depression and anxiety.^33^ In line with this, we analyzed the factor structure and internal consistency of the PHQ-SADS as a composite measure for depression (PHQ-9), anxiety (GAD-7), and also somatization (PHQ-15) in our sample (eMethods 1). Based on this analysis, we used the sum of 27 relevant items as primary outcome.

Self-reported secondary outcomes were posttraumatic symptoms (Posttraumatic Stress Disorder Checklist for DSM-5 (PCL-5)^34,35^), insomnia (Insomnia Severity Index (ISI)^36,37^), unhealthy alcohol use (Alcohol Use Disorders Identification Test (AUDIT)^38^), repetitive negative thinking (Perseverative Thinking Questionnaire (PTQ)^39^), mental well-being (Mental Health Continuum – Short Form (MHC-SF)^40^), posttraumatic growth (Posttraumatic Growth Inventory – Short Form (PTGI-SF)^41^), mindfulness (Five Facet Mindfulness Questionnaire – Short Form (FFMQ-SF)^42^), and self-compassion (Self-Compassion Scale – Short Form (SCS-SF)^43^).

All measures were administered at baseline, post-intervention, and 3- and 6-month follow-up.

Halfway through both interventions and at post-intervention, participants were asked to indicate how often they did mindfulness exercises per week and how many minutes they spent on average per exercise. The mean time spent on mindfulness per week during the intervention period was calculated; for the adjusted MBSR, the time spent on video conferencing sessions was also included. Winsorizing^44^ was used to handle outliers (defined as *z*-score above 3.29) reported in frequency and duration of mindfulness exercises and replaced by the highest score not being an outlier (mean + 2xSD).

The study coordinator registered any serious adverse events reported by participants and informed the independent institutional review board, and weekly monitored suicidal ideation (PHQ-9) at all assessments points.

### Statistical Analysis

Sample size calculation was based on detecting an intervention*time interaction between both interventions at 6-month follow-up with a medium effect size (Cohen’s *d*=0.40; alpha=0.05; power=80%). Anticipating 10% attrition, the targeted sample size was 220 (110 per arm). A statistical analysis plan was preregistered before data collection was completed (https://doi.org/10.17605/OSF.IO/W4A2P).

The primary analysis was an intention-to-treat analysis. Linear mixed effect modelling was used to test our primary hypothesis. Group assignment was included as fixed effect, and we controlled for baseline PHQ-SADS. Cohort (*n*=10) was added as a random effect. Cohen’s *d* effect size between both groups was calculated by dividing the adjusted group difference at 6-month follow-up by the pooled standard deviation at baseline.^45^

To evaluate the course of mental health symptoms from baseline to 6-month follow-up, linear mixed effects models were used with restricted maximum likelihood estimation to handle missing data. Time, group assignment, and their interaction were added as fixed effects. A random effect for participant was added. Cohort was not added as a random factor, due to the small interclass correlation (ICC=0.015) in the primary analysis. A heterogeneous first-order autoregressive (ARH(1)) covariance structure was used. Post hoc pairwise comparisons within-groups were adjusted for multiple comparisons using the Bonferroni method. Cohen’s *d* effect size within-group was calculated for the timeframes from baseline to each follow-up measurement.^45^

Sensitivity analyses were conducted by performing primary and secondary analyses in a per-protocol sample. The per-protocol analysis sample consisted of HCWs who had a minimum attendance of four adjusted MBSR sessions. Moderation was examined by adding the potential moderator and its interaction with group to the model. Separate models were run for each possible moderator (gender, age, work setting, profession, prior mindfulness training, adherence, preference for intervention, baseline scores of mindfulness, self-compassion, perseverative thinking). In addition, an exploratory post hoc analysis was done to examine the effect of dose of mindfulness practice during both interventions on the PHQ-SADS by adding dose, group assignment, and their interaction to the primary model.

Statistical analyses were performed with the IBM SPSS Statistics Version 27 program.

## RESULTS

### Participants

Between August 2020 and September 2021, 201 participants were randomly assigned to either the adjusted MBSR (*n*=99) or the self-guided MBI (*n*=102). Participants were predominantly women (*n*=189; 94%); 47 (23.4%) worked as physicians, 120 (59.7%) as nurses, and 34 (16.9%) as supporting staff (e.g., technicians in medical imaging, spiritual counsellors, physiotherapists). Table 1 summarizes baseline characteristics of participants. Of 66 approached Dutch hospitals, 28 (40%) were willing to recurrently share the study information with their employees. Ten cohorts started between August 2020 and September 2021; the median number of HCWs in the adjusted MBSR was 11 (range 6 to 13). 83 of 99 HCWs in the adjusted MBSR participated in four or more sessions, of which 69 attended seven or eight sessions. The latest follow-up assessment was in June 2022. Of the 201 participants, 19 (9.5%) were lost to follow-up. Of these, nine were in the adjusted MBSR. Those who were lost to follow-up reported fewer working years in healthcare and higher baseline posttraumatic symptoms than those who completed the assessments (eTable 2). In total, 182 full cases were included instead of the required 198.

**Table 1:** Baseline Characteristics of the Intention-to-Treat Population, by Intervention Group

	Adjusted MBSR *N*= 99	Self-guided MBI *N*= 102
Age (years), mean (SD)	40.6 (12.0)	40.7 (11.9)
Gender		
Female	92 (92.9)	97 (95.1)
Male	7 (7.1)	5 (4.9)
Married	46 (46.5)	46 (45.1)
Children	54 (54.5)	62 (60.8)
Education		
Post-secondary non-tertiary	16 (16.2)	22 (21.6)
Bachelor or equivalent	55 (55.6)	50 (49.0)
Master/doctoral or equivalent	28 (28.3)	30 (29.4)
Setting		
Hospital	74 (74.7)	73 (71.6)
Nursing home	10 (10.1)	12 (11.8)
Other	15 (15.2)	17 (16.7)
Profession		
Physician, Physician assistant	23 (23.2)	24 (23.5)
Nurse, Nurse anesthetist	60 (60.6)	60 (58.8)
Other	16 (16.2)	18 (17.6)
Working years in healthcare, mean (SD)	13.6 (10.8)	13.6 (12.4)
Current psychological treatment	20 (20.2)	25 (24.5)
Previous psychological treatment	52 (52.5)	56 (54.9)
Prior 8-week mindfulness training	11 (11.1)	11 (10.8)
Preference intervention		
Adjusted MBSR	36 (36.4)	37 (36.3)
Self-guided MBI	24 (24.2)	26 (25.5)
None	39 (39.4)	39 (38.2)
Scores at baseline, mean (SD)		
PHQ-9	7.7 (4.5)	7.2 (4.3)
GAD-7	7.1 (4.3)	6.3 (4.0)
PHQ-15	8.3 (4.4)	7.0 (3.9)
PHQ-SADS	20.2 (10.7)	17.7 (9.4)

Values are numbers (%) unless stated otherwise. *GAD*, generalized anxiety disorder; *MBI*, mindfulness-based intervention; *MBSR*, mindfulness-based stress reduction; *PHQ*, patient health questionnaire; *SADS*, somatic, anxiety, and depressive symptom scales

### Primary Outcome at 6-Month Follow-up

At 6-month follow-up, no difference in the combined depressive, anxiety, and somatic symptoms was found between the adjusted MBSR and the self-guided MBI (mean difference (SE) PHQ-SADS, 0.23 (1.03); *P*=0.82; Cohen’s *d* (95% CI) 0.023 (−0.253; 0.300)).

### Secondary Outcomes

**Figure 2:**
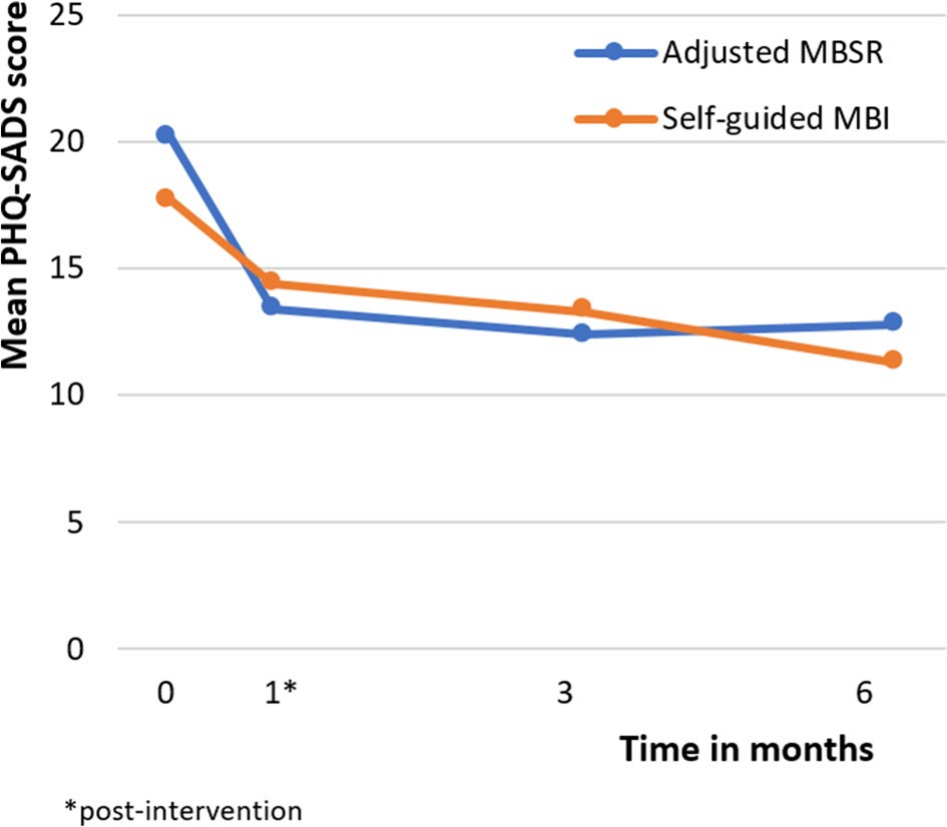
Mean Patient Health Questionnaire – Somatic Anxiety Depressive Symptom Scales (PHQ-SADS) scores over time in the two interventions.

Psychological symptoms (PHQ-SADS) significantly decreased over time in both groups (Tables 2 and 3; Fig. 2). The course of decrease in PHQ-SADS differed between both interventions, evidenced by a significant interaction effect between intervention and time (*F*(3, 420)=3.99, *P*=0.008). Post hoc analyses within-group showed a greater reduction of the PHQ-SADS from baseline to post-intervention in the adjusted MBSR than in the self-guided MBCI (Cohen’s *d* effect size between baseline and post-intervention for adjusted MBSR −0.71 (95% CI −1.01; −0,42), for self-guided MBI −0.35 (95% CI −0.63; −0.06)) (Table 3).

**Table 2:** Intention-to-Treat Results for Outcomes in Adjusted MBSR and Self-guided MBI

				Change across all time points			
Outcomes (score range)*		Adjusted MBSR *N*= 99	Self-guided MBI *N*= 102				
		Mean (SD)	Mean (SD)		*F*	*df*	*P* value
PHQ-SADS (0–**70**)	T0	20.20 (10.66)	17.71 (9.40)	*Intervention*	0.28	209	0.60
	T1	13.30 (8.10)	14.40 (9.66)	*Time*	30.91	419	< 0.001
	T2	11.78 (8.31)	13.27 (9.04)	Intervention*Time	3.99	419	< 0.01
	T3	12.49 (8.22)	11.30 (8.37)				
PCL- 5 (0–**80**)	T0	17.74 (12.56)	15.44 (11.38)	*Intervention*	0.25	204	0.62
	T1	11.28 (11.15)	12.07 (10.75)	*Time*	20.78	423	< 0.001
	T2	9.74 (10.26)	10.16 (8.89)	Intervention*Time	2.30	423	0.08
	T3	10.04 (10.05)	9.04 (8.84)				
ISI (0–**28**)	T0	15.92 (6.20)	15.42 (5.74)	*Intervention*	0.16	204	0.69
	T1	13.93 (5.76)	13.68 (5.11)	*Time*	14.39	437	< 0.001
	T2	13.04 (5.05)	13.30 (4.74)	Intervention*Time	0.31	437	0.82
	T3	13.00 (5.08)	12.55 (3.93)				
AUDIT (0–**40**)	T0	3.36 (3.21)	3.78 (2.79)	*Intervention*	1.84	197	0.18
	T1	2.63 (2.28)	3.44 (2.85)	*Time*	8.10	485	< 0.001
	T2	2.82 (2.36)	3.17 (2.55)	Intervention*Time	1.01	485	0.39
	T3	2.66 (2.19)	3.05 (2.53)				
PTQ (0–**60**)	T0	29.70 (11.85)	28.04 (10.51)	*Intervention*	0.90	200	0.35
	T1	24.59 (11.08)	24.14 (10.78)	*Time*	26.63	430	< 0.001
	T2	24.41 (10.02)	24.02 (10.26)	Intervention*Time	0.53	430	0.66
	T3	23.01 (11.03)	21.49 (9.79)				
MHC-SF (**0**–5)	T0	2.92 (0.93)	3.07 (0.84)	*Intervention*	0.92	203	0.34
	T1	3.33 (0.95)	3.28 (0.96)	*Time*	15.96	387	< 0.001
	T2	3.31 (0.94)	3.36 (0.95)	Intervention*Time	1.45	387	0.23
	T3	3.33 (0.98)	3.46 (0.89)				
PTGI-SF (**0**–50)	T0	27.48 (9.70)	27.68 (8.92)	*Intervention*	6.57	204	0.01
	T1	31.91 (11.56)	26.41 (10.30)	*Time*	2.32	350	0.08
	T2	31.13 (11.26)	27.26 (11.09)	Intervention*Time	5.32	350	0.001
	T3	31.48 (11.04)	27.49 (11.00)				
FFMQ-SF (**24**–120)	T0	77.56 (11.58)	79.24 (11.93)	*Intervention*	0.64	206	0.43
	T1	82.69 (12.46)	83.14 (11.87)	*Time*	23.76	412	< 0.001
	T2	83.41 (11.75)	83.72 (11.84)	Intervention*Time	0.84	412	0.47
	T3	84.42 (12.10)	86.33 (12.64)				
SCS-SF (**1**–7)	T0	3.97 (1.08)	3.99 (1.12)	*Intervention*	0.26	204	0.61
	T1	4.51 (1.23)	4.51 (1.11)	*Time*	32.51	426	< 0.001
	T2	4.56 (1.06)	4.63 (1.09)	Intervention*Time	0.68	426	0.57
	T3	4.61 (1.12)	4.76 (1.10)				

*AUDIT*, Alcohol Use Disorders Identification Test; *ISI*, Insomnia Severity Index; *FFMQ-SF*, Five Facet Mindfulness Questionnaire–Short Form; *MBI*, Mindfulness-based intervention; *MBSR*, Mindfulness-based Stress Reduction; *MHC-SF*, Mental Health Continuum–Short Form; *PCL-5*, Posttraumatic Stress Disorder Checklist for DSM-5; *PTGI-SF*, Posttraumatic Growth Inventory–Short Form; *PTQ*, Perseverative Thinking Questionnaire

^*^The bold number of the score range represents the worst score

**Table 3:** Post Hoc Analyses for Secondary Outcomes in Adjusted MBSR and Self-guided MBI

	Post hoc analyses*				
		Adjusted MBSR		Self-guided MBI	
		Mean difference between timepoints (SE)	Within-group effect size, Cohen’s ***d*** (95% CI)	Mean difference between timepoints (SE)	Within-group effect size, Cohen’s ***d*** (95% CI)
PHQ-SADS	T0–T1	− 6.82 (0.93)^†^	− 0.71 (− 1.01; − 0.42)	− 3.30 (0.78)^†^	− 0.35 (− 0.63; − 0.06)
	T0–T2	− 7.82 (1.15)^†^	− 0.81 (− 1.01; − 0.50)	− 4.45 (0.96)^†^	− 0.48 (− 0.77; − 0.19)
	T0–T3	− 7.46 (1.24)^†^	− 0.78 (− 1.07; − 0.48)	− 6.45 (1.04)^†^	− 0.72 (− 1.01; − 0.43)
PCL- 5	T0–T1	− 6.39 (1.11)^†^	− 0.54 (− 0.83; − 0.24)	− 3.02 (0.95)^‡^	− 0.27 (− 0.55; 0.01)
	T0–T2	− 7.75 (1.34)^†^	− 0.67 (− 0.97; − 0.37)	− 5.11 (1.11)^†^	− 0.50 (− 0.79; − 0.21)
	T0–T3	− 7.23 (1.48)^†^	− 0.63 (− 0.92; − 0.34)	− 6.39 (1.23)^†^	− 0.62 (− 0.91; − 0.34)
ISI	T0–T1	− 2.02 (0.52)^†^	− 0.34 (− 0.63; − 0.04)	− 1.55 (0.49)^‡^	− 0.28 (− 0.57; 0.001)
	T0–T2	− 2.50 (0.64)^†^	− 0.44 (− 0.73; − 0.14)	− 1.95 (0.55)^‡^	− 0.37 (− 0.66; − 0.08)
	T0–T3	− 2.85 (0.71)^†^	− 0.50 (− 0.79; − 0.21)	− 2.80 (0.58)^†^	− 0.56 (− 0.85; − 0.28)
AUDIT	T0–T1	− 0.65 (0.18)^‡^	− 0.23 (− 0.52; 0.05)	− 0.41 (0.15)^§^	− 0.15 (− 0.43; 0.13)
	T0–T2	− 0.520 (0.23)	− 0.18 (− 0.48; 0.11)	− 0.59 (0.20)^§^	− 0.22 (− 0.50; 0.07)
	T0–T3	− 0.72 (0.26)^§^	− 0.26 (− 0.55; 0.03)	− 0.73 (0.23)^‡^	− 0.27 (− 0.56; 0.001)
PTQ	T0–T1	− 5.00 (0.88)^†^	− 0.44 (− 0.72; − 0.14)	− 3.77 (0.80)^†^	− 0.35 (− 0.65; − 0.08)
	T0–T2	− 4.81 (1.01)^†^	− 0.44 (− 0.73; − 0.14)	− 4.19 (1.00)^†^	− 0.40 (0.79; − 0.12)
	T0–T3	− 6.39 (1.27)^†^	− 0.56 (− 0.85; − 0.27)	− 6.58 (1.12)^†^	− 0.65 (0.94; − 0.36)
MHC-SF	T0–T1	0.39 (0.07)^†^	0.42 (0.12; 0.71)	0.22 (0.07)^‡^	0.25 (− 0.04; 0.53)
	T0–T2	0.31 (0.09)^‡^	0.33 (0.04; 0.63)	0.31 (0.09)^‡^	0.35 (0.06; 0.64)
	T0–T3	0.40 (0.10)^†^	0.42 (0.13; 0.71)	0.41 (0.10)^†^	0.48 (0.19; 0.76)
PTGI-SF	T0–T1	4.41 (1.07)^†^	0.42 (0.12; 0.71)	− 1.06 (0.91)	− 0.11 (− 0.39; 0.17)
	T0–T2	3.30 (1.25)^§^	0.33 (0.03; 0.63)	− 0.25 (1.17)	− 0.02 (− 0.31; 0.26)
	T0–T3	3.80 (1.32)^§^	0.37 (0.08; 0.66)	− 0.19 (1.28)	− 0.02 (− 0.30; 0.26)
FFMQ-SF	T0–T1	5.21 (0.93)^†^	0.43 (0.15; 0.73)	3.68 (0.82)^†^	0.31 (0.02; 0.29)
	T0–T2	5.30 (1.17)^†^	0.45 (0.16; 0.75)	4.53 (1.07)^†^	0.38 (0.09; 0.67)
	T0–T3	6.56 (1.34)^†^	0.55 (0.27; 0.85)	6.91 (1.27)^†^	0.56 (0.27; 0.84)
SCS-SF	T0–T1	0.54 (0.11)^†^	0.47 (0.17; 0.75)	0.51 (0.10)^†^	0.46 (0.17; 0.75)
	T0–T2	0.54 (0.12)^†^	0.50 (0.20; 0.80)	0.64 (0.12)^†^	0.58 (0.29; 0.87)
	T0–T3	0.62 (0.13)^†^	0.56 (0.27; 0.85)	0.75 (0.13)^†^	0.68 (0.39; 0.97)

*AUDIT*, Alcohol Use Disorders Identification Test; *ISI*, Insomnia Severity Index; *FFMQ-SF*, Five Facet Mindfulness Questionnaire–Short Form; *MBI*, Mindfulness-based intervention; *MBSR*, Mindfulness-based Stress Reduction; *MHC-SF*, Mental Health Continuum–Short Form; *PCL-5*, Posttraumatic Stress Disorder Checklist for DSM-5; *PTGI-SF*, Posttraumatic Growth Inventory–Short Form; *PTQ*, Perseverative Thinking Questionnaire

Cohen’s *d* effect sizes were corrected for sample size

^*^Post hoc analyses using pairwise comparison within-group with Bonferroni correction

^†^*P*< 0.001; ^‡^*P*< 0.01; ^§^*P*< 0.05

For both interventions, a similar within-group reduction of PHQ-SADS symptoms at 6-month follow-up was observed with medium to large effect sizes (Cohen’s *d* between baseline and 6-month follow-up: adjusted MBSR −0.78 (95% CI −1.07; −0.48), self-guided MBI −0.72 (95% CI −1.01; −0.43)).

The trajectory of posttraumatic growth differed between both interventions (intervention*time *F*(3, 350)=5.32, *P*=0.001). Post analyses within-group showed an exclusive increase in posttraumatic growth at post-intervention in the adjusted MBSR with a sustaining effect in the follow-up period and not in the self-guided MBI (Cohen’s *d* effect size between baseline and post-intervention for adjusted MBSR 0.42 (95% CI 0.12; 0.71), for self-guided MBI −0.11 (95% CI −0.39; 0.17)). Analyses of all other secondary outcomes revealed no between-group differences. However, significant within-group reductions were found for insomnia, PTSS symptoms, and repetitive negative thinking in both groups, with medium effect sizes (Tables 2 and 3). Furthermore, within-group increases in positive mental health, mindfulness, and self-compassion had small to medium effect sizes (Tables 2 and 3).

### Additional Analyses

Results of per-protocol analysis were comparable with the results of the intention-to-treat analysis for both primary and secondary outcomes (eTables 3 and 4).

None of the possible moderators influenced the relationship between intervention and PHQ-SADS at 6-month follow-up.

An exploratory analysis showed a dose-response relation of more practice during the intervention period accompanied by larger improvements in PHQ-SADS (*F*(1, 166)=7.793, *P*=0.006), irrespective of adjusted MBSR or self-guided MBI (group*practice (*F*(1, 166)=0.54, *P*=0.46)). For each additional weekly practice increase of 1 h, an additional decrease of 1.4 points on the PHQ-SADS can be expected. See eTable 5 for results on time spent on mindfulness practice.

### Adverse Events

During follow-up, 11 HCWs spontaneously reported adverse events, one in the adjusted MBSR (burnout), and ten in the self-guided MBI (one recurrent depression; two illness due to COVID-19; six burnout; one diagnosis of idiopathic hypersomnia). Two participants in the self-guided MBI reported frequent suicidal ideation during follow-up assessments and were advised to contact their general practitioner.

## DISCUSSION

This RCT comparing adjusted therapist-assisted MBSR group intervention versus minimal self-guided MBI in a mixed group of frontline HCWs during the COVID-19 pandemic showed no superiority of the adjusted MBSR over the self-guided MBI: both interventions were accompanied by substantial within-group reductions in depressive, anxiety, and somatic symptoms at 6-month follow-up. The largest reduction in both interventions was observed from just before to immediately after the 4-week intervention with consolidation of this effect during the follow-up period. The adjusted MBSR showed a greater reduction of psychological symptoms at 1 month (post-intervention) than the self-guided MBI. Furthermore, both interventions showed similar reductions of posttraumatic stress, insomnia, and repetitive negative thinking at 6-month follow-up. Mindfulness skills and self-compassion increased as well as mental well-being.

Obviously, the lack of a treatment as usual or waitlist control group precludes absolute conclusions about the effectiveness of both mindfulness-based intervention. However, the largest effect was seen pre-to-post intervention. Together with the dose-response relationship, this is pointing toward a relation with the interventions, as otherwise a more gradual effect over time would be expected for HCWs. Moreover, longitudinal non-interventional studies in COVID-19 HCWs demonstrated persistence of psychological symptoms and overall poor mental health.^5,46,47^ If a natural recovery of psychological symptoms is reported, this is usually more gradual.^4,6^ A recent RCT (*n*=232) on an evidence-based stepped-care program including two crisis interventions for HCWs reported a fast and lasting reduction of anxiety and depression in the intervention groups versus a smaller gradual decrease in the control group^15^, a very similar pattern to our study.

In the light of the recent meta-analysis on web-based mindfulness interventions for HCWs during the COVID-19 pandemic^23^, our study supports that MBIs positively contribute to the mental health of HCWs. We are the first to provide sufficient long-term follow-up data, and compare two different MBIs in a large sample of a variety of frontline HCWs from different healthcare institutions. Based on the low drop-out rates in our study and the high adherence to the adjusted MBSR, the commitment to the interventions seemed high, despite the large time investment required. This might be related to the amount of suffering and the lack of other support. We experienced more problems in getting hospitals to disseminate the study information. Surprisingly, the format of the interventions did not make a major difference. Our self-guided MBI was tailored to the crisis situation, but not further personalized. This self-guided format might address concerns around confidentiality, privacy, and stigma^48^, and benefit from relatively low cost and high scalability. However, an individually delivered, therapist-assisted online version of MBI, live or asynchronous, might be even more effective than the current format. Furthermore, future qualitative research is needed to investigate the barriers and facilitators of different possible formats of online MBIs to support implementation.

An additional observation is that only HCWs in the adjusted MBSR reported an increase in posttraumatic growth at post-intervention, the experience of positive change that occurs as a result of the struggle with highly challenging life crises.^49^ An explanation for this difference could be the peer support and the guidance of a qualified teacher facilitated in the adjusted MBSR. Perceived social support during the COVID-19 pandemic was associated with posttraumatic growth^50^, and sharing experiences in a congenial group may help to incorporate new perspectives and transcend individual stories.^49^

### Limitations

Besides the lack of an inactive control group, there are some limitations relevant for discussion. Our study was slightly underpowered, mainly due to the difficulties we experienced in recruiting potential participants when the COVID-19 crisis gradually subsided. Furthermore, our recruitment methods unfortunately prevented us to know how large our target population was. Due to unbalanced groups (e.g., gender) in the moderation analysis, the null findings should be interpreted with caution. In addition, almost all participants were women (*n*= 189; 94.0%), which largely corresponds to the gender distribution among HCWs.^51,52^ This limits the generalizability of our findings to male HCWs.

In conclusion, we observed no difference in psychological symptoms between the adjusted therapist-assisted MBSR group intervention and the minimal self-guided MBI for frontline HCWs during the COVID-19 pandemic. However, both interventions showed substantial and comparable within-group reductions of psychological symptoms at 6-month follow-up. At post-intervention, a greater reduction of these psychological symptoms and exclusive increase of posttraumatic growth was observed in the adjusted MBSR. On the other hand, the self-guided MBI might be more accessible and easily scalable. Given the current capacity problems in healthcare and the high probability of future healthcare crises, and in absence of a usual care comparator, we recommend to further investigate the possible role of MBIs to support frontline HCWs to maintain psychological health under extreme pressure such as the COVID-19 pandemic.

## Supplementary Information

Below is the link to the electronic supplementary material.

Supplementary file1 (DOCX 51.7 KB )

## Data Availability

Individuals’ participant data that underlie the results reported in this article (after de-identification) will be available on request. After reviewing the quality of the request, permission will be granted if the request is in accordance with the terms of use drafted by the Radboud University Medical Center. An embargo period of 6 months after publication will be applied. The study protocol, statistical analysis plan, and informed consent form will also be provided on request.
