# Supplementary material for: Mindfulness-Based Interventions for Mental Health Outcomes in Frontline Healthcare Workers During the COVID-19 Pandemic: A Randomized Controlled Trial
Source: J Gen Intern Med. 2025 May 19;40(15):3705–14. doi: 10.1007/s11606-025-09529-z (PMC12612424; doi:10.1007/s11606-025-09529-z)
Supplement: Supplementary file 1 — Supplementary file1 (DOCX 51.7 KB ) [file 11606_2025_9529_MOESM1_ESM.docx]

**Supplementary Information**

**eTable 1.** Description of both mindfulness-based interventions

**eMethods 1.** Confirmatory factor analysis

**eTable 2.** Baseline characteristics by lost to follow-up

**eTable 3.** Per protocol results for secondary outcomes in adjusted MBSR and self-guided MBI

**eTable 4**. Post hoc analyses of per protocol results for secondary outcomes in adjusted MBSR and self-guided MBI

**eTable 5.** Time spent on mindfulness practice in adjusted MBSR and self-guided MBI

**eTable 1. Description of both Mindfulness-based Interventions**

| **Adjusted therapist-assisted MBSR group intervention** | **Minimal self-guided mindfulness-based intervention** |
| --- | --- |
| ***WEEK 1*** | |
| **SESSION 1: Automatic pilot (90 min)**  Welcome, introduction of trainer(s) (5 min)  Introduction on mindfulness and compassion (10 min)  Intention meditation and introductory round (20 min)  Introduction on training and ground rules (10 min)  Moment to stretch/get a raison (3 min)  Raison exercise (15 min)  Bodyscan + sharing experiences (20 – 5 min)  Information about use of booklet and home practice next week (5 min) | DAY 1: Compassion: acknowledgment of suffering in yourself and others, and responding compassionately (27 min)  [Exercise: Awareness of breathing and kindness meditation](https://www.youtube.com/watch?v=bobj0_-1Z8o&amp;t=14s) |
|  | DAY 2: Autopilot versus awareness (27 min)  [Exercise: Short body scan](https://www.youtube.com/watch?v=X3VonkECQbs) |
|  | DAY 3: Audio meditation basic training (41 min)  [Exercise: Body scan](https://www.youtube.com/watch?v=PHldew8fbLQ&amp;t=8s) |
| **SESSION 2: Seeing clearly – thoughts are not facts**  Welcome (5 min)  Window watching exercise and debriefing (15 min)  Bodyscan and discussion of current experiences in twosomes (20 – 10 min)  Inquiry bodyscan and home practice (20 min)  Exercise ‘Walking down the street’ (15 min)  Home practice next week, sitting with focus on breath ending with ‘What do you wish for yourself’ (10 min) | DAY 4: Selfcare 1: While caring for others, how do you take care of yourself? (29 min)  [Exercise: Awareness of breathing and 3-minute breathing space](https://www.youtube.com/watch?v=fYMovvFowfQ&amp;t=2s) |
|  | DAY 5: Dwell on pleasant experience (30 min)  [Exercise: Self compassion](https://www.youtube.com/watch?v=DRGJA7EwftE) |
|  | **DAY 6: Audio meditation basic training (44 min)** [Exercise: Lying movement exercises](https://www.youtube.com/watch?v=hbQs8KniGOI&amp;t=4s) |
| ***WEEK 2*** | |
| **SESSION 3: From doing to being**  Welcome (5 min)  Movement exercises (sitting) emphasis on compassion for the body (25 min)  Inquiry exercises and home practice (20 min)  Short sitting exercise focus on pleasant experience, sharing in twosomes (10 min)  Group discussion pleasant experience exercise, triangle of awareness (20 min)  Introducing 3 minute breathing space, including ‘How can I best take care of myself right now?’ (10 min)  Home practice next week (1 min) | DAY 1: Recognizing stress reactions (27 min)  [Exercise: Sitting meditation, focus on breath and bodily sensations](https://www.youtube.com/watch?v=UAisNPu6ztI) |
|  | DAY 2: Audio meditation basic training (41 min)  [Exercise: Body scan](https://www.youtube.com/watch?v=PHldew8fbLQ&amp;t=8s) |
|  | DAY 3: Dealing with fear and uncertainty (28 min)  [Exercise: Sitting meditation, focus on breath, bodily sensations and sounds](https://www.youtube.com/watch?v=1-VDVCMHlFA) |
| **SESSION 4: Being present**  Welcome (1 min)  Sitting meditation (20 min)  Inquiry sitting meditation and home practice (15 min)  Standing movement exercises (10 min)  Short sitting exercise focus on unpleasant experience, sharing in twosomes (15 min)  Discussing unpleasant experience exercise in group. Triangle of awareness, making connection with own patterns/standard reactions (25 min)  3 minute breathing space with compassion, home practice next week (5 min) | DAY 4: Audio meditation basic training (44 min)  [Exercise: Lying movement exercises](https://www.youtube.com/watch?v=hbQs8KniGOI&t=4s) |
|  | DAY 5: Dealing with difficult emotions (28 min)  [Exercise: Softening, soothing, allowing](https://www.youtube.com/watch?v=6QDtFC4VVzw) |
|  | **DAY 6: Being with suffering (31 min)** [Exercise: Sitting meditation, focus on breath, bodily sensations and sounds](https://www.youtube.com/watch?v=dnRCFEm5yiM) |
| ***WEEK 3*** | |
| **SESSION 5: Responding to stress rather than reacting**  Welcome (1 min)  Guided exercise ending with question “How is it going until now?”, sharing on what is changing and how you want to continue with commitment (20 min)  Short sitting exercise focus on stressful event, sharing in twosomes (15 min)  Group discussion, paying attention on reaction versus response (20 min)  Standing movement exercises (5 min)  Exercise ‘Softening, soothing, allowing’ (15 min)  Inquiry exercise (10 min), ending with poem ‘Autobiography in 5 captures’  3 minute breathing space, home practice next week (5 min) | DAY 1: Acknowledging the suffering in yourself and others and how we can alleviate it (30 min)  [Exercise: Kindness meditation](https://www.youtube.com/watch?v=MYHuBJQE1yU) |
|  | **DAY 2: Audio meditation basic training (45 min)**  [Exercise: standing movement exercises](https://www.youtube.com/watch?v=T44ZkdLfPmo&t=1435s) |
|  | DAY 3: Dealing with stress, from reaction to response (25 min)  [Exercise: Mountain meditation](https://www.youtube.com/watch?v=4SR5xjSNcog) |
| **SESSION 6: Communication with awareness**  Welcome (1 min)  Sitting meditation (20 min)  Inquiry sitting meditation and home practice (15 min)  Movement exercises (sitting or standing) (15 min)  Short sitting practice with reflection on difficult communication. Sharing in twosomes, taking turns on speaking and listening (15 min)  Group discussion on communication exercise (15 min)  3 minute breathing space, home practice next week, information on optional silence daypart (10 min) | DAY 4: Audio meditation basic training (38 min)  [Exercise: Sitting meditation](https://www.youtube.com/watch?v=RUnArisoa2c&t=133s) |
|  | DAY 5: Dealing with difficult circumstances (26 min)  [Exercise: Self compassion](https://www.youtube.com/watch?v=ER8s7yEOwec) |
|  | **DAY 6: Communication in stressful times (26 min)** [Exercise: Sitting with a difficulty](https://www.youtube.com/watch?v=BFR7j-CzDxo) |
| ***WEEK 4*** | |
| **SESSION 7: Life in balance**  Welcome (1 min)  Kindness meditation (25 min)  Inquiry mediation and home practice/silence daypart (15 min)  Movement exercises (5 min)  Exercise on activities taking or giving energy; guiding day and writing down (15 min)  Reflection on exercise in twosomes, encourage action plan (10 min)  Group discussion (15 min)  3 minute breathing space, home practice next week (5 min) | DAY 1: Tackling the internal critic (27 min)  [Exercise: Kindness meditation](https://www.youtube.com/watch?v=V2OsScnmcQ0) |
|  | **DAY 2: Audio meditation basic training (32 min)**  [Exercise: Sitting movement exercises](https://www.youtube.com/watch?v=p82REHCYcRc) |
|  | DAY 3: Dealing with restlessness (27 min)  [Exercise: Lake meditation](https://www.youtube.com/watch?v=6CfV11PtHzM&t=3s) |
| **SESSION 8: The eight week will continue for the rest of your life**  Welcome (1 min)  Compassionate bodyscan and short reflection (25 min)  Sharing action plan in twosomes (10 min)  Sitting meditation, ending with last 5 minutes exploring what the training brought you and what you want to remember (15 min)  Group evaluation: What have you learned? How will you continue? (30 min)  Directing to information about how to continue (10 min)  3 minute breathing space, silence, reading a poem (5 min) | DAY 4: Energizers and energy consumers (25 min)  [Exercise: Awareness for the breath and body, 3-minute breathing space](https://www.youtube.com/watch?v=DyCZ2BqLH5A) |
|  | DAY 5: Audio file meditation basic training (37 min)  [Exercise: Sitting mediation](https://www.youtube.com/watch?v=RUnArisoa2c&t=133s) |
|  | **DAY 6: Generosity and gratitude (33 min)** [Exercise: Compassionate body scan](https://www.youtube.com/watch?v=O1gqG-ioGkQ) |
| **OPTIONAL SILENCE DAYPART: Practicing independently at home with audio files**  Standing exercises (40 min)  Short bodyscan (20 min)  Tea or coffee break in silence (10 min)  Sitting meditation (20 min)  Walking meditation: walking outside in silence with focus on senses  Kindness meditation (20 min) |  |

**eMethods 1.** Confirmatory factor analysis of composite measure PHQ-SADS

Previous studies using confirmatory factor analysis revealed sufficient unidimensionality to support the use of the total score of the PHQ-SADS as a composite measure of depression and anxiety.^1^ In line we analyzed the factor structure and internal consistency of the PHQ-SADS as a composite measure for depression (PHQ-9), anxiety (GAD-7) and also somatization (PHQ-15) in our sample. We removed four items of the PHQ-15: two of them were identical to PHQ-9 items (item 14 and 15), one was only relevant for women (pain or problems with menstruation) and one only for people in a sexual relationship (pain or problems with intercourse). A principal factor analysis (PCA) of the remaining items revealed seven principal components with an Eigenvalue greater than one. The scree test showed one large factor left to the elbow of the graph with an Eigenvalue of 8.22 explaining 30.45% of the variance. Factor loadings of the 27 items varied from .34 - .77 with only four loadings < .40. The internal consistency of the scale consisting of these 27 items was excellent (Cronbach's alpha=.91). Consequently, we used the sum of these 27 items as a composite measure for depression, anxiety and somatization as primary outcome.

1. Kroenke K, Wu J, Yu Z, et al. Patient Health Questionnaire Anxiety and Depression Scale: Initial Validation in Three Clinical Trials. *Psychosom Med*. Jul-Aug 2016; 78(6):716-27.

**eTable 2. Baseline characteristics by lost to follow-up**

|  | **Lost to follow-up** | |  |
| --- | --- | --- | --- |
|  | No  N=182 | Yes  N=19 |  |
| Age, mean (SD), y | 41.0 (12.0) | 37.1 (10.4) | t_23_=1.56; *P* = 0.13 |
| Female | 170 (93.4) | 19 (100) | *P* = 0.61 |
| Married | 85 (46.7) | 7 (36.8) | *P* = 0.47 |
| Children | 108 (59.3) | 8 (42.1) | *P* = 0.22 |
| Education |  |  | *P* = 0.62 |
| Post-secondary non-tertiary | 36 (19.8) | 2 (10.5) |  |
| Bachelor or equivalent | 93 (51.1) | 12 (63.2) |  |
| Master/doctoral or equivalent | 53 (29.1) | 5 (26.3) |  |
| Work setting |  |  | *P*= 0.19 |
| Hospital | 133 (73.1) | 14 (73.7) |  |
| Nursing home | 18 (9.9) | 4 (21.0) |  |
| Other | 31 (17.0) | 1 (5.3) |  |
| Profession |  |  | *P*= 0.44 |
| Physician, Physician assistant | 42 (23.1) | 5 (26.3) |  |
| Nurse, Nurse anesthetist | 107 (58.8) | 13 (68.4) |  |
| Other | 33 (18.1) | 1 (5.3) |  |
| Working years in healthcare, mean (SD), y | 14.2 (11.7) | 8.3 (9.0) | *t*_25_=2.60; *P*= 0.02 |
| Current psychological treatment | 42 (23.5) | 3 (16.7) | *P*= 0.77 |
| Previous psychological treatment | 97 (53.3) | 11 (57.9) | *P*= 0.81 |
| Prior 8-week mindfulness training | 20 (11.0) | 2 (10.5) | *P*= 1.00 |
| Preference intervention |  |  | *P*= 0.36 |
| Adjusted MBSR | 69 (37.9) | 4 (21.0) |  |
| Self-guided MBI | 44 (24.2) | 6 (31.6) |  |
| None | 69 (37.9) | 9 (47.4) |  |
| Scores at baseline, mean (SD) |  |  |  |
| PHQ-SADS | 18.7 (10.3) | 21.0 (7.3) | *t*_26_=-1.20; *P*= 0.24 |
| PCL-5 | 16.1 (12.2) | 21.4 (9.8) | *t*_24_=-2.18; *P*= 0.04 |
| ISI | 15.5 (5.9) | 16.8 (6.2) | *t*_22_=-1.02; *P*= 0.32 |
| AUDIT | 3.55 (2.94) | 3.84 (3.7) | *t*_21_=-0.40; *P*= 0.74 |
| PTQ | 28.7 (11.2) | 30.1 (11.4) | *t*_22_=-0.48; *P*= 0.63 |
| MHC-SF | 3.00 (0.88) | 2.95 (0.96) | *t*_21_=0.18; *P*= 0.86 |
| PTGI | 27.5 (9.1) | 28.3 (11.1) | *t*_21_=-0.31; *P*= 0.76 |
| FFMQ-SF | 78.5 (11.8) | 77.3 (11.2) | *t*_22_=0.42; *P*= 0.68 |
| SFS-SF | 4.00 (1.09) | 3.81 (1.19) | *t*_21_=0.64; *P*= 0.53 |
| Values are numbers (%) unless otherwise stated. AUDIT=Alcohol Use Disorders Identification Test; ISI=Insomnia Severity Index; FFMQ-SF=Five Facet Mindfulness Questionnaire–Short Form; MBI=Mindfulness-based intervention; MBSR=Mindfulness-based Stress Reduction; MHC-SF=Mental Health Continuum–Short Form; PCL-5=Posttraumatic Stress Disorder Checklist for DSM-5; PTGI-SF=Posttraumatic Growth Inventory–Short Form; PTQ=Perseverative Thinking Questionnaire; All categorical data were tested with the Fisher Exact Test; All means were compared with Levene’s Test. | | | |

**eTable 3. Per protocol results for secondary outcomes in adjusted MBSR and self-guided MBI**

|  |  |  |  |  |  | **Per protocol analyses** | | | |
| --- | --- | --- | --- | --- | --- | --- | --- | --- | --- |
|  |  |  | **Adjusted MBSR** |  | **Self-guided MBI** |  |  |  |  |
|  |  | n | Mean (SD) | n | Mean (SD) |  | F | *df* | *P* value |
| PHQ-SADS | T0 | 83 | 19.24 (10.67) | 102 | 17.71 (9.40) | *Intervention* | 0.00 | 192 | 0.99 |
|  | T1 | 80 | 12.81 (7.71) | 90 | 14.40 (9.66) | *Time* | 27.26 | 404 | <0.001 |
|  | T2 | 76 | 11.84 (8.04) | 88 | 13.27 (9.04) | *Intervention*Time* | 3.54 | 404 | 0.02 |
|  | T3 | 80 | 12.27 (8.29) | 91 | 11.30 (8.37) |  |  |  |  |
| PCL-5 | T0 | 83 | 16.94 (13.14) | 102 | 15.44 (11.38) | *Intervention* | 0.02 | 189 | 0.90 |
|  | T1 | 80 | 11.02 (11.21) | 90 | 12.07 (10.75) | *Time* | 17.60 | 407 | <0.001 |
|  | T2 | 76 | 9.61 (9.76) | 88 | 10.16 (8.89) | *Intervention*Time* | 2.04 | 407 | 0.11 |
|  | T3 | 80 | 10.01 (10.37) | 93 | 9.04 (8.84) |  |  |  |  |
| ISI | T0 | 83 | 15.66 (6.24) | 102 | 15.42 (5.74) | *Intervention* | 0.03 | 189 | 0.86 |
|  | T1 | 80 | 13.45 (5.42) | 90 | 13.68 (5.11) | *Time* | 14.17 | 415 | <0.001 |
|  | T2 | 76 | 13.01 (4.83) | 88 | 13.30 (4.74) | *Intervention*Time* | 0.46 | 415 | 0.71 |
|  | T3 | 80 | 12.74 (4.95) | 93 | 12.55 (3.93) |  |  |  |  |
| AUDIT | T0 | 83 | 3.33 (3.09) | 102 | 3.78 (2.79) | *Intervention* | 1.60 | 184 | 0.21 |
|  | T1 | 80 | 2.73 (2.32) | 90 | 3.44 (2.85) | *Time* | 6.99 | 463 | <0.001 |
|  | T2 | 76 | 2.89 (2.41) | 87 | 3.17 (2.55) | *Intervention*Time* | 0.98 | 463 | 0.40 |
|  | T3 | 80 | 2.71 (2.22) | 92 | 3.05 (2.53) |  |  |  |  |
| PTQ | T0 | 83 | 29.01 (10.91) | 102 | 28.04 (10.51) | *Intervention* | 0.15 | 187 | 0.70 |
|  | T1 | 79 | 24.00 (10.47) | 90 | 24.14 (10.78) | *Time* | 26.96 | 409 | <0.001 |
|  | T2 | 74 | 24.31 (10.08) | 87 | 24.02 (10.26) | *Intervention*Time* | 0.83 | 409 | 0.48 |
|  | T3 | 80 | 22.49 (10.63) | 91 | 21.49 (9.79) |  |  |  |  |
| MHC-SF | T0 | 83 | 2.98 (0.94) | 102 | 3.07 (0.84) | *Intervention* | 0.20 | 187 | 0.66 |
|  | T1 | 80 | 3.37 (0.95) | 90 | 3.28 (0.96) | *Time* | 14.55 | 375 | <0.001 |
|  | T2 | 76 | 3.32 (0.94) | 87 | 3.36 (0.95) | *Intervention*Time* | 1.75 | 375 | 0.16 |
|  | T3 | 80 | 3.34 (0.98) | 93 | 3.46 (0.89) |  |  |  |  |
| PTGI-SF | T0 | 83 | 27.83 (9.83) | 102 | 27.68 (8.92) | *Intervention* | 7.21 | 191 | <0.01* |
|  | T1 | 79 | 31.80 (10.99) | 90 | 26.41 (10.30) | *Time* | 2.18 | 341 | 0.09 |
|  | T2 | 74 | 30.57 (10.83) | 87 | 27.26 (11.09) | *Intervention*Time* | 5.36 | 341 | 0.001 |
|  | T3 | 80 | 31.30 (10.34) | 91 | 27.49 (11.00) |  |  |  |  |
| FFMQ-SF | T0 | 83 | 77.59 (11.36) | 102 | 79.24 (11.93) | *Intervention* | 0.56 | 190 | 0.46 |
|  | T1 | 80 | 82.79 (11.62) | 90 | 83.14 (11.87) | *Time* | 22.03 | 397 | <0.001 |
|  | T2 | 75 | 83.13 (11.22) | 87 | 83.72 (11.84) | *Intervention*Time* | 1.01 | 397 | 0.39 |
|  | T3 | 80 | 84.21 (11.68) | 91 | 86.33 (12.64) |  |  |  |  |
| SCS-SF | T0 | 83 | 4.02 (1.06) | 102 | 3.99 (1.12) | *Intervention* | 0.01 | 188 | 0.92 |
|  | T1 | 80 | 4.51 (1.17) | 90 | 4.51 (1.11) | *Time* | 29.98 | 408 | <0.001 |
|  | T2 | 76 | 4.55 (1.02) | 87 | 4.63 (1.09) | *Intervention*Time* | 0.60 | 408 | 0.61 |
|  | T3 | 80 | 4.68 (1.11) | 92 | 4.76 (1.10) |  |  |  |  |
| AUDIT=Alcohol Use Disorders Identification Test; ISI=Insomnia Severity Index; FFMQ-SF=Five Facet Mindfulness Questionnaire–Short Form; MBI=Mindfulness-based intervention; MBSR=Mindfulness-based Stress Reduction; MHC-SF=Mental Health Continuum–Short Form; PCL-5=Posttraumatic Stress Disorder Checklist for DSM-5; PTGI-SF=Posttraumatic Growth Inventory–Short Form; PTQ=Perseverative Thinking Questionnaire; *Mean difference in PTGI-SF between groups at 6-month follow-up 3.105 (SE 1.21), Cohen’s d effect size 0.33. | | | | | | | | | |

**eTable 4. Post hoc analyses of per protocol results for secondary outcomes in adjusted MBSR and self-guided MBI**

|  | **Post hoc analyses^*^** | | | | |
| --- | --- | --- | --- | --- | --- |
|  |  | Adjusted MBSR | | Self-guided MBI | |
|  |  | Mean difference between timepoints (SE) | Within-group  effect size, Cohen’s *d* (95% CI) | Mean difference between timepoints (SE) | Within-group effect size, Cohen’s *d* (95% CI) |
| PHQ-SADS | T0 – T1 | -6.53 (0.98)† | -0.70 (-1.02;-0.38) | -3.30 (0.78)† | -0.35 (-0.63;-0.06) |
|  | T0 – T2 | -7.03 (1.22)† | -0.74 (-1.06;-0.42) | -4.45 (0.96)† | -0.48 (-0.77;-0.19) |
|  | T0 – T3 | -6.85 (1.34)† | -0.72 (-1.03;-0.40) | -6.45 (1.04)† | -0.72 (-1.01;-0.43) |
| PCL-5 | T0 – T1 | -6.03 (1.19)† | -0.49 (-0.80;-0.18) | -3.02 (0.95)‡ | -0.27 (-0.55;0.01) |
|  | T0 – T2 | -7.23 (1.44)† | -0.62 (-0.94;-0.30) | -5.11 (1.11)† | -0.50 (-0.79;-0.21) |
|  | T0 – T3 | -6.94 (1.63)† | -0.56 (-0.87;-0.25) | -6.39 (1.23)† | -0.62 (-0.91;-0.34) |
| ISI | T0 – T1 | -2.28 (0.57)† | -0.39 (-0.67;-0.08) | -1.55 (0.49)‡ | -0.28 (-0.57;0.001) |
|  | T0 – T2 | -2.41 (0.69)† | -0.45 (-0.76;-0.13) | -1.95 (0.55)‡ | -0.37 (-0.66;-0.08) |
|  | T0 – T3 | -2.95 (0.77)† | -0.52 (-0.83;-0.21) | -2.80 (0.58)† | -0.56 (-0.85;-0.28) |
| AUDIT | T0 – T1 | -0.58 (0.19)‡ | -0.21 (-0.52;0.10) | -0.41 (0.15)§ | -0.15 (-0.43;0.13) |
|  | T0 – T2 | -0.42 (0.25) | -0.15 (-0.46;0.16) | -0.59 (0.20)§ | -0.22 (-0.50;0.07) |
|  | T0 – T3 | -0.64 (0.28) | -0.24 (-0.54;0.07) | -0.73 (0.23)‡ | -0.27 (-0.56;0.001) |
| PTQ | T0 – T1 | -5.31 (0.90)† | -0.49 (-0.80;-0.18) | -3.77 (0.80)† | -0.35 (-0.65;-0.08) |
|  | T0 – T2 | -4.74 (1.14)† | -0.45 (-0.76;-0.13) | -4.19 (1.00)† | -0.40 (0.79;-0.12) |
|  | T0 – T3 | -6.36 (1.32)† | -0.61 (-0.93;-0.30) | -6.58 (1.12)† | -0.65 (0.94;-0.36) |
| MHC-SF | T0 – T1 | 0.40 (0.08)† | 0.43 (0.12;0.74) | 0.22 (0.07)‡ | 0.25 (-0.04;0.53) |
|  | T0 – T2 | 0.30 (0.10)‡ | 0.32 (0.01;0.63) | 0.31 (0.09)‡ | 0.35 (0.06;0.64) |
|  | T0 – T3 | 0.37 (0.11)‡ | 0.38 (0.07;0.69) | 0.41 (0.10)† | 0.48 (0.19;0.76) |
| PTGI-SF | T0 – T1 | 4.52 (1.11)† | 0.43 (0.12-0.75) | -1.06 (0.91) | -0.11 (-0.39;0.17) |
|  | T0 – T2 | 3.02 (1.33) | 0.29 (-0.02;0.61) | -0.25 (1.17) | -0.02 (-0.31;0.26) |
|  | T0 – T3 | 3.43 (1.41)§ | 0.34 (0.03;0.65) | -0.19 (1.28) | -0.02 (-0.30;0.26) |
| FFMQ-SF | T0 – T1 | 5.42 (1.01)† | 0.47 (0.16;0.78) | 3.68 (0.82)† | 0.31 (0.02;0.29) |
|  | T0 – T2 | 5.32 (1.27)† | 0.47 (0.15;0.79) | 4.53 (1.07)† | 0.38 (0.09;0.67) |
|  | T0 – T3 | 6.49 (1.46)† | 0.56 (0.25;0.88) | 6.91 (1.27)† | 0.56 (0.27;0.84) |
| SCS-SF | T0 – T1 | 0.54 (0.09)† | 0.48 (0.17;0.79) | 0.51 (0.10)† | 0.46 (0.17;0.75) |
|  | T0 – T2 | 0.52 (0.11)† | 0.50 (0.19;0.82) | 0.64 (0.12)† | 0.58 (0.29;0.87) |
|  | T0 – T3 | 0.63 (0.13)† | 0.58 (0.27;0.89) | 0.75 (0.13)† | 0.68 (0.39;0.97) |
| AUDIT=Alcohol Use Disorders Identification Test; ISI=Insomnia Severity Index; FFMQ-SF=Five Facet Mindfulness Questionnaire–Short Form; MBI=Mindfulness-based intervention; MBSR=Mindfulness-based Stress Reduction; MHC-SF=Mental Health Continuum–Short Form; PCL-5=Posttraumatic Stress Disorder Checklist for DSM-5; PTGI-SF=Posttraumatic Growth Inventory–Short Form; PTQ=Perseverative Thinking Questionnaire; Cohen’s *d* effect sizes were corrected for sample size; * Post hoc analyses using pairwise comparison within-group with Bonferroni correction; † *P*< 0.001; ‡ *P*< 0.01; § *P*< 0.05. | | | | | |

**eTable 5. Time spent on mindfulness practice in adjusted MBSR and self-guided MBI**

|  | Adjusted MBSR | | | | Self-guided MBI | | | |
| --- | --- | --- | --- | --- | --- | --- | --- | --- |
|  | *Time spent on mindfulness practice at home* | | | | | | | |
|  | N | Frequency per week | Duration in minutes | Time practice per week in minutes* | N | Frequency per week | Duration in minutes | Time practice per week in minutes* |
| T0 – Tmid | 87 | 4.8 (2.5) | 23.3 (13.5) | 117.6 (96.9) | 90 | 4.2 (1.7) | 29.0 (12.0) | 130.2 (72.4) |
| Tmid – T1 | 87 | 3.1 (2.1) | 19.7 (12.1) | 67.7 (66.3) | 90 | 3.0 (1.9) | 23.1 (13.1) | 79.1 (67.9) |
|  | *Time spent on video conferencing videos* | | | | | | | |
| T0 – T1 | 99 |  |  | 139.1 (52.8) |  |  |  |  |
| Values are expressed in mean (SD). Winsorizing was used to handle outliers (defined as z-score above 3.29) reported in frequency and duration of mindfulness exercises and replaced by the highest score not being an outlier (mean + 2xSD). MBI=Mindfulness-based intervention; MBSR=Mindfulness-based Stress Reduction; Tmid=measure halfway intervention. *Time practice per week in minutes was calculated by frequency per week x duration in minutes, when one of the measures at Tmid or T1 was missing, the available value was extrapolated to the missing value. | | | | | | | | |
